# Empyema Tube or No Tube?

**DOI:** 10.7759/cureus.12829

**Published:** 2021-01-20

**Authors:** Bhoobalan Magendiran, Stalin Viswanathan, Jayachandran Selvaraj, Vivekanandan Pillai

**Affiliations:** 1 General Medicine, Jawaharlal Institute of Postgraduate Medical Education and Research, Pondicherry, IND

**Keywords:** spontaneous bacterial empyema, hepatic hydrothorax, empyema

## Abstract

We report the case of a 36-year-old man with cirrhosis who presented with recurrent infection of his right-sided hepatic hydrothorax in the form of fever, dyspnea, and cough. The pleural fluid analysis showed transudative fluid with normal pH, lactic acid dehydrogenase, and glucose, but with *Escherichia coli* growth. An uncommon diagnosis of high mortality, spontaneous bacterial empyema was made. Criteria for chest tube drainage were met, but he was managed without one. He developed hospital-acquired pneumonia during his stay, but his pleural fluid showed the same characteristics. His empyema and pneumonia were managed with antibiotics and other supportive measures. On follow-up, he was readmitted on three other occasions with similar complaints and succumbed to upper gastrointestinal bleed during the fifth admission. A chest tube is not indicated in patients with spontaneous bacterial empyema unless frank pus is present.

## Introduction

The term spontaneous bacterial empyema (SBEM) is used to define an infection of hepatic hydrothorax without the evidence for other causes of infection in the pleural space or lung parenchyma [1]. It has been described in 13-16% of patients with hepatic hydrothorax [1]. This is an under-diagnosed entity as thoracocentesis is not routinely employed in the above patients. Flaum first described this entity in a 48-year-old man who had *Escherichia coli*-related SBEM [2]. The criteria for the diagnosis of SBEM were framed by Xiol et al. in their study of eight patients [3]. We describe a 36-year-old man with recurrent SBEM whose second admission was complicated with hospital-acquired pneumonia, but the pleural fluid continued to fulfill the criteria for SBEM. Traditional indications for tubal drainage, such as growth in the pleural fluid, were met, but he was managed conservatively with antibiotics alone.

## Case presentation

**Figure 1:**
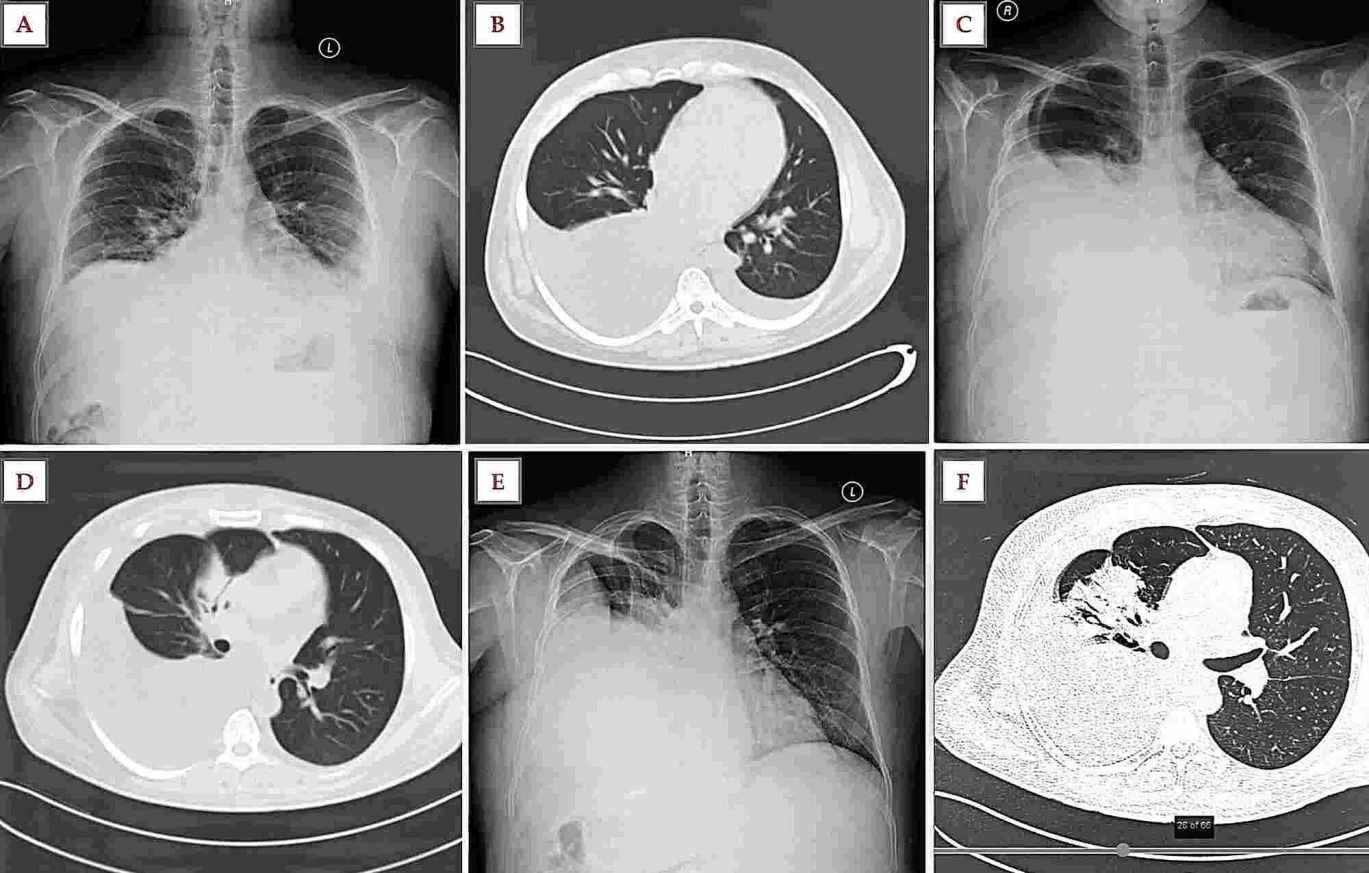
Imaging during the first two admissions. (A) Chest radiography (following thoracocentesis) during the previous admission shows mild left-sided pleural effusion and right-sided costophrenic angle blunting. (B) CT chest during the previous admission revealing bilateral effusion (R>L). (C and D) Chest radiography and CT chest at admission (current) showing right-sided pleural effusion. (E) Chest radiography on day nine shows right-sided moderate-to-submassive pleural effusion (following three sittings of therapeutic thoracocentesis). (F) CT chest on day nine reveals a right-sided pleural effusion and right middle lobe pneumonia. CT, computed tomography

A 36-year-old male with alcoholic cirrhosis (Child-Pugh score 11) for the preceding eight months presented with fever and dyspnea of 15 days duration; dry cough and melena were present for the last five days. His workup for Wilson disease, viral hepatitis, and autoimmune causes was inconclusive. He had grade 2 esophageal varices. He had quit alcohol 18 months ago. He had been admitted two months earlier with complaints of fever and dyspnea and a right-sided pleural effusion (Figure 1A, 1B). At admission, he was tachypneic (24 breaths/min), tachycardic (118 beats/min), normotensive with massive splenomegaly, and features suggestive of massive right-sided pleural effusion (Figure 1C). He did not have ascites, jaundice, encephalopathy, or coagulopathy.

Diagnostic and therapeutic thoracentesis was performed (thrice over seven days, each time draining approximately 500 mL). Pleural fluid was transudative, with 6,700 leukocytes/hpf (N90% L10%), glucose 100 mg/dL, pH 7.41, adenosine deaminase of 20 IU/L, and grew *E. coli *on the third day of admission (Table 1). Pleural fluid acid-fast bacilli (AFB) and GeneXpert were negative. Computed tomography (CT) of the thorax showed a massive right-sided pleural effusion (Figure 1D). Serum alfa fetoprotein and prostate-specific antigen were within normal ranges.

**Table 1:** Biochemical and hematological investigations. WBC, white blood cells; LDH, lactate dehydrogenase

	Previous Admission	Current Admission		
Day		Day 1	Day 9	At discharge
Hemoglobin (g/dL)	8.2	9.0	8.0	7.5
WBC (x10^9^/L)	7.65	5.00	2.33	6.47
Platelets (x10^9^/L)	48	75	62	56
Reticulocytes (%)	-	-	2.5	2.2
Total bilirubin (mg/dL)	6.24	4.07	4.1	4.7
Direct bilirubin (mg/dL)	2.44	1.96	1.92	1.91
Aspartate transaminase (IU/L)	60	95	76	60
Alanine transaminase (IU/L)	25	21	20	16
Urea (mg/dL)	30	11	13	12
Creatinine (mg/dL)	0.85	0.62	0.59	0.55
Pleural fluid				
Counts (/hpf)	5,340	6,700	2,320	400
Differentials (%)	N90 L10	N90 L10	N75 L25	N75 L25
Protein (g/dL)	0.9	0.6	0.6	0.6
Albumin (g/dL)	0.3	0.2	0.1	0.2
Serum-pleural albumin gradient	1.6	1.5	1.4	1.8
Glucose (mg/dL)	132	100	140	122
pH	-	7.41	7.36	7.42
LDH (U/L)	70	82	109	50
Gram stain	-	Negative	-	-
Culture	-	E. coli	-	-

His previous admission records showed bicytopenia (Table 1), splenomegaly without ascites, normal echocardiography and electrocardiogram, normal renal and thyroid function tests, and mildly deranged liver function tests. Diagnostic and therapeutic (800 mL) thoracentesis showed a similar transudative pleural effusion with elevated neutrophil counts that were negative for AFB, pancreatitis, and malignancy. CT thorax revealed normal lung parenchyma and bilateral pleural effusion (Figure 1B). The patient had been treated conservatively with ceftriaxone, frusemide, sodium and fluid restriction, and vitamins with which he showed improvement and was discharged home.

Considering the immunocompromised state, fever, elevated neutrophil counts in the pleural fluid, and a positive bacterial culture, a diagnosis of empyema or a complicated parapneumonic effusion was kept, except that the pH (>7.2), glucose (>40 mg/dL), and lactate dehydrogenase (<1,000 U/L) did not match either diagnosis and rather fit the criteria of a simple parapneumonic effusion.

Piperacillin/Tazobactam 4.5 gQ6H was empirically initiated at admission. Initially, because of a positive culture, a four-week antibiotic course was planned, and chest tube drainage was considered for empyema. This was deferred in view of previous hydrothorax and a similar pleural infection and thrombocytopenia. Following a diagnosis of SBEM, only a conservative approach was pursued. Antibiotics were stepped down to ceftriaxone following a review of records. On the seventh day of admission, bronchial breath sounds were heard in the right infra-scapular areas, followed by fever (39.7 °C) and delirium on day nine. A repeat chest radiograph (Figure 1E) followed by a CT chest (Figure 1F) revealed consolidation in the right middle lobe. Levofloxacin 500 mg was initiated, with which he became afebrile in 48 hours and sensorium was normalized. Intravenous albumin 80 g was administered once, and levofloxacin was given for seven days. Repeat thoracentesis showed a sterile fluid with 400 cells/hpf (N75 L25%), following which he was discharged on day 18 with a diagnosis of SBEM.

Follow-up

**Figure 2:**
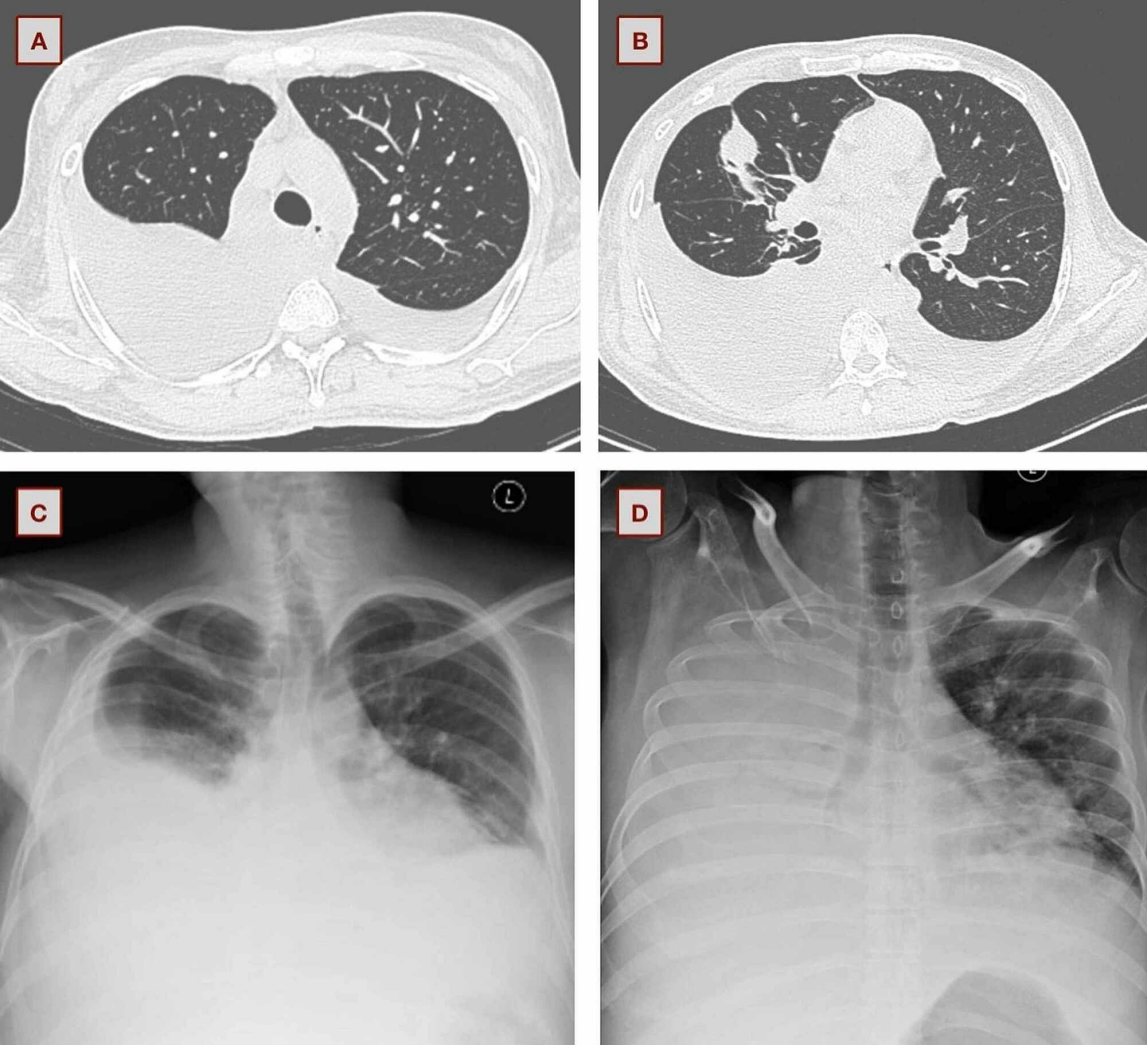
Chest imaging during his subsequent admissions. (A and B) Bilateral pleural effusion (R>L) during the third admission. (C) Moderate right-sized effusion during his fourth admission. (D) Massive right-sided pleural effusion at the fifth admission.

The patient remained well and had no similar complaints when he followed up two months later. Two and a half months later, he presented to the hospital again with respiratory distress and hepatic encephalopathy. Ascitic fluid (cells: 5,630, neutrophils: 90%) and pleural fluid (cells: 2,360, neutrophils: 89%) revealed spontaneous bacterial peritonitis (SBP) and SBEM, respectively. Radiology revealed bilateral pleural effusion (R>L) (Figure 2A, 2B). He was given ceftriaxone for two weeks, along with anti-hepatic coma therapy. Five months later, he presented with fever and breathlessness. Moderate pleural effusion was noted (Figure 2C). Pleural fluid and blood culture grew *E. coli *sensitive to amikacin, which he received for 10 days. Seven months after discharge, he was readmitted with SBP (cells: 50,000, neutrophils: 90%) and SBEM (cells: 39,130, neutrophils: 90%) associated with tense ascites and massive pleural effusion, respectively (Figure 2D). *E. coli* sensitive to amikacin, piperacillin-tazobactam, and cefoperazone-sulbactam was cultured. He received antibiotics and multiple therapeutic paracentesis and thoracentesis without improvement in respiratory distress. The course was complicated by upper gastrointestinal bleed and shock to which he succumbed.

## Discussion

Hepatic hydrothorax refers to the presence of a pleural effusion (usually >500 mL) in a patient with cirrhosis after excluding other causes of pleural effusion (cardiac, pulmonary, or pleural disease) [4]. Hepatic hydrothorax occurs in approximately 5-10% of patients with cirrhosis. In cirrhotic patients, the prevalence of SBEM is <2%, while a quarter of patients with hepatic hydrothorax can develop SBEM [5]. Just as SBP is seen in patients with ascites, patients with hepatic hydrothorax may develop SBEM. It is thought to occur due to seeding of the pleural effusion with organisms that spread directly from the abdominal cavity in a patient with SBP or from bacteremia [6]. Spontaneous bacterial empyema is diagnosed when a patient with hepatic hydrothorax presents with fever, respiratory symptoms, or encephalopathy, and the pleural fluid analysis shows mostly a transudative fluid, a positive fluid culture, and a polymorphonuclear (PMN) cell count of >250 cells/μL without evidence of pneumonia on chest imaging. A negative pleural fluid culture should have a PMN cell count of >500 cells/μL. [6]. The most frequent organisms are of the *Enterobacteriaceae *family, namely, *E coli* and *Klebsiella pneumoniae*, as seen in our patient [1]. Third-generation cephalosporins are the drugs of choice in SBEM as in SBP [1]. Empirically, considering a possibility of a healthcare-associated parapneumonic effusion, piperacillin-tazobactam had been initiated at admission. Following the review of his previous records and recent use of ceftriaxone, piperacillin-tazobactam was continued. For possible extended-spectrum beta-lactamases producing organisms with previous cephalosporin use (<three months), carbapenems may be necessary [1]. Additionally, levofloxacin was administered in view of hospital-acquired pneumonia. Despite his pneumonia, the pleural fluid continued to remain transudative and without an increase in the size of the effusion. Empyema necessitates drainage of pleural fluid [7]. Tubal drainage in SBEM is indicated only in the presence of frank pus [1].

## Conclusions

We report a case of recurrent empyema not requiring tube drainage, although traditional criteria of empyema were fulfilled. A review of previous records, the patient’s predisposing illness, and mismatch of empyema criteria with the fluid counts and protein are suggestive clues to shorten the duration of antibiotic therapy and need avoidance of a chest tube in such patients. Emergency and respiratory physicians need to be aware of disorders in their practice.
